# Integrated proteomics analysis and network pharmacology to elucidate the mechanism of Zhilong Huoxue Tongyu Capsule alleviate hypertensive retinopathy in Ang II infusion mice model

**DOI:** 10.3389/fphar.2025.1573693

**Published:** 2025-05-21

**Authors:** Jiao Wu, Wen Xie, Yucen Xie, Maryam Mazhar, Junguo Duan

**Affiliations:** ^1^ Eye School of Chengdu University of TCM, Chengdu University of Traditional Chinese Medicine, Chengdu, China; ^2^ Department of Cardiology, Affiliated Hospital of Chengdu University of Traditional Chinese Medicine, Chengdu, China; ^3^ National Traditional Chinese Medicine Clinical Research Base and Drug Research Center, The Affiliated Traditional Chinese Medicine Hospital of Southwest Medical University, Luzhou, China; ^4^ Ineye Hospital of Chengdu University of Traditional Chinese Medicine, Chengdu, China; ^5^ Key Laboratory of Sichuan Province Ophthalmopathy Prevention & Cure and Visual Function Protection with TCM Laboratory, Chengdu, China

**Keywords:** ZhiLong HuoXue TongYu Capsule, traditional Chinese medicine, hypertensive retinopathy, NLRP3 inflammasome, pyroptosis

## Abstract

**Background:**

Zhilong Huoxue Tongyu Capsule (ZLHXTY) has been used in clinical treatment of vascular diseases caused by hypertension over 20 years. However, the specific mechanisms by ZLHXTY alleviate hypertensive retinopathy (HR) needs to be further explored.

**Materials and methods:**

HR mouse model was established by infusing Ang II via subcutaneously implanted osmotic mini-pumps, followed by oral administration of ZLHXTY (0.35, 0.7, 1.4 g/kg/day) 28 days for treatment. To assess the impacts of ZLHXTY on retinal neurodegeneration and vascular injury, multiple experiments such as OCTA, ERG and HE staining were performed. Subsequently, network pharmacological and 4D-label-free proteomics to clarify the potential targets and mechanisms of ZLHXTY alleviated HR. Finally, Western blot, ELISA, IF, and other techniques were utilized to detect the expression of proteins related to inflammation, oxidative stress, and NLRP3 inflammasome activation.

**Results:**

ZLHXTY significantly alleviated retinal dysfunction, increased retinal blood flow, and mitigated pathological changes such as retinal tissues edema in HR mice. Network pharmacology indicated that ZLHXTY might exert anti-inflammatory and anti-oxidative stress effects through targets such as TNF and NF-κB. Proteomic analysis showed that the differential proteins between the ZL group and the Ang II group were mainly enriched in the immune-inflammatory response, and the main mechanism of which might be related to the assembly of NLRP3 inflammasome. Subsequent *in vivo* experiments corroborated that ZLHXTY remarkably attenuated inflammation and oxidative stress damage in retinal tissues. Further experiments demonstrated that ZLHXTY inhibited the NLRP3/Caspase-1/GSDMD signaling pathway and related protein expression. Finally, TEM results also verified that ZLHXTY alleviated pyroptosis in retinal cells.

**Conclusion:**

Our results suggest that ZLHXTY by regulating the NLRP3/Caspase-1/GSDMD axis, inhibiting pyroptosis, thereby relieving retinal dysfunction and vascular injury in HR mice.

## 1 Introduction

Hypertension is a prevalent and leading causes of cardiovascular diseases, which exhibits high morbidity and elevated all-cause mortality (Kario et al., 2024). Chronic elevation of blood pressure leads to arteriosclerotic changes in vascular system including ocular vasculature, leading to hypertensive retinopathy (HR) (Alswailmi, 2023). This condition disrupts both the retinal nerve fiber layer and retinal microcirculation, resulting in significant retinal damage (Dziedziak et al., 2022). It is commonly exhibited in 76.6% of the patients with primary hypertension (Li et al., 2023). It is usually asymptomatic in earlier stages, while, visual field defects and blurred vision occur in the later stages of the disease (Dziedziak et al., 2022). Untreated hypertension can lead to irreversible vision loss acutely, either due to retinal pigmentary alteration following exudative retinal detachment or secondary optic atrophy following long-term papilloedema (Do et al., 2023). Nevertheless, researches have not well elucidated the molecular mechanisms underlying HR.

The innate immune system is the primary defense against pathogens and can crucially maintain homeostasis (Cheng et al., 2024). NOD-like receptor pyrin domain-containing 3 (NLRP3) is an innate immune receptor capable of recognizing pathogen-associated molecular patterns and damage-associated molecular patterns, promoting inflammasome to be formed (Kuo et al., 2022). The NLRP3 inflammasome encompasses the sensor protein NLRP3, the adaptor protein apoptosis-associated speck-like protein (ASC), and the effector protease caspase-1 (Kodi et al., 2024). NLRP3 inflammasome activation triggers pro-inflammatory cytokines (IL-1β and IL-18) to be secreted, thereby mediating the inflammatory response (Yang et al., 2019). According to previous studies, NLRP3 upregulation is positively correlated with inflammation in HR mice, while, NLRP3 inhibition makes pro-inflammatory cytokines less secreted (Li J. et al., 2024). Nowadays, there has been more focus given to study the interaction between NLRP3 inflammasome and pyroptosis, a type of lytic programmed cell death (Coll et al., 2022). The NLRP3 inflammasome converts pro-caspase-1 into active caspase-1, cleaving the C-terminal of GSDMD and generating several N-terminal (GSDMD-NT) active spliceosome (Vande Walle and Lamkanfi, 2024). These GSDMD-NT spliceosome migrate to the cell membrane, inducing cellular perforation. As a result, cell death is triggered and massive inflammatory substances are released (Zheng et al., 2023). Pyroptosis is related to the pathogenesis with regard to several retinal diseases (age-related macular degeneration, diabetic retinopathy, and retinal pigment epithelial degeneration) (Al et al., 2021). According to recent studies, spontaneously hypertensive rats present elevated NLRP3 and caspase-1 (Li Y. et al., 2024). On this account, pyroptosis possibly exerts a significant effect on HR. However, the precise mechanism by which NLRP3 inflammasome induced pyroptosis contributes to HR remains unclear.

Zhilong Huoxue Tongyu Capsule (ZLHXTY) is a type of traditional Chinese medicine (TCM, Patent No. 200810147774.1) enjoying a wide application in treating hypertension-associated vascular diseases (Liu et al., 2021; Zhao X. et al., 2024). The formulation primarily encompasses Astragalus membranaceus Fisch Bunge, Sargentodoxa cuneate (Oliv.) Rehd and E. H.Wilson, Cinnamomum cassia (L.) J. Presl, Hirudo nipponica Whitman, Pheretima Aspergillum (E.Perrier). Table 1 lists the specific information. The efficacy of ZLHXTY in treating hypertensive vascular diseases is supported by numerous randomized controlled trials. Several clinical studies demonstrate that ZLHXTY can improve atherosclerosis, coronary heart disease, cerebral infarction (Liu et al., 2021), and other peripheral vascular diseases, particularly HR. A review indicates that ZLHXTY primarily exerts its effects by alleviating arterial wall sclerosis and thickening, reducing lumen narrowing, and regulating lipid and blood rheology abnormalities (Liang et al., 2021). In addition, extensive basic research demonstrates that ZLHXTY can improve endothelial cell dysfunction (Bi et al., 2024), alleviate endothelial cell pyroptosis (Liu et al., 2022), and inhibit neuronal apoptosis (Geng et al., 2023). From the perspective of TCM, HR and other vascular diseases are considered similar, both attributed to Qi deficiency and blood stasis. The effective treatment strategies involve tonifying Qi, promoting blood circulation, dispelling wind and resolving stasis. ZLHXTY is a treatment developed according to the theory of Xuanfu to invigorating qi, expelling wind, activating blood and dissolving stasis. It adheres to the principles of syndrome differentiation and treatment in TCM. Mechanistically, Inhibition of inflammation and oxidative stress are hot spots in HR treatment. Interestingly, antioxidant and anti-inflammatory properties are common to ZLHXTY ingredients (Liang et al., 2021). Thus, we speculated that ZLHXTY could alleviates HR.

**TABLE 1:** The detail information of Zhilong Huoxue Tongyu capsule.

Chinese name	Latin name	Drug name	Medicalnal part	Dose (g)	Vocher specimen number
Huang Qi	Astragalus membranaceus Fisch. ex Bunge	Astragalus	Dried Roots	2.3	90802
Di long	Pheretima Aspergillum (E.Perrier)	Earthworm	Dried whole animal	1.7	190301
Shui zhi	Hirudo nipponica Whitman	Leech	Dried whole animal	0.32	190401
Da xue teng	Sargentodoxa cuneate (Oliv.) Rehd. Et Wils	Sargentgloryvine	Dried Twig	1.7	190402
Gui zhi	Cinnamomum cassia(L.)J.Presl	Cassia	Dried Twig	0.86	190801

## 2 Materials and methods

### 2.1 Preparation of ZLHXTY

ZLHXTY were obtained from Affiliated TCM Hospital of Southwest Medical University (Luzhou, China). All herbs components had been authenticated by Prof. Qingrong Pu, director of the TCM Preparation Room at the same institution. The processing and preparation of the ingredients were carried out at the pharmacy department of the Hospital (Luzhou, China). Briefly, the herbs were soaked in water (1:12) for half an hour, followed by half an hour of boiling. The process was repeated three times. After filtration, a rotary evaporator was used to concentrate the combined filtrates, which underwent vacuum freeze drying to obtain a fine powder of ZLHXTY. In previous study by our group, ZLHXTY underwent ultrahigh-performance liquid chromatography electrospray ionization orbitrap tandem mass spectrometry (UPLC-HR-MS) analysis (Bi et al., 2024).

### 2.2 Reagents and antibodies

Angiotensin II (Ang II, A9525) was purchased from Sigma (Darmstadt, Germany). Enalapril (H32026567) was purchased from Yangzijiang Pharmaceutical (Taizhou, China). Pentobarbital sodium salt (P3761) was purchased from Sigma-Aldrich (shanghai, China). Compound Tropicamide Eye Drops (H20123453) was purchased from Shenyang Xingqi Pharmaceutical Co., Ltd. (Shenyang, China). Haematoxylin-Eosin kit (G1120) was purchased from SOLARBIO (Beijing, China). ELISA kits (IL-6:P1326, IL-18:P1553, IL-1β:P1301) was purchased from Beyotime Biotechnology (Shanghai, China). The kits of MDA (A003-1-2), GSH (A006-2-1), GSH-px (A005-1-2), SOD (A001-3-2) and CAT (A007-1-) were all purchased from Nanjing Jiancheng Bioengineering Institute (Nanjing, China). The One Step TUNEL Apoptosis Assay Kit (C1089), the DHE Kit (S0064S), BCA protein assay kit (BL521A), RIPA buffer (BL504A), BeyoECL Plus kit (P0018S) were purchased from Beyotine Biotechnology (Shanghai, China). Antibodie includes Iba-1 (ab5076), Goat Anti-Rabbit IgG H&L (Alexa Fluor^®^ 594) (ab150080), Goat Anti-Rabbit IgG H&L (Alexa Fluor^®^ 488) (ab150077) were sourced from Abcam (MA, United States). The Caspase 1 antibody (22915-1-AP), VEGFA antibody (81323-2-RR), GFAP antibody (16825-1-AP), TNF-α antibody (17590-1-AP), GSDMD antibody (20770-1-AP), NF-κB p65 antibody (10745-1-AP), IL-1 beta antibody (26048-1-AP), GAPDH antibody (10494-1-AP), goat anti-rabbit IgG antibody (b900210) were all sourced from Proteintech (Wuhan, China). The NeuN Rabbit mAb (A19086), NLRP3 Rabbit pAb (A5652) were sourced from Abclonal (Wuhan, China).

### 2.3 Animals

Male C57BL/6J mice (n = 75, 6–8 weeks old) came from Chengdu Gembio (SCXK 2022-034, Chengdu, China). All animals were raised in specific pathogen-free conditions, maintained at 22°C ± 2°C with 12-h light/dark cycle, and with free access to water and food. This study obtained the approval of the Animal Research Committee of Southwest Medical University, Luzhou, Sichuan, China (Approval No: 20241108-006). The HR model was established by subcutaneously infusing Ang II (3,000 ng/kg/min), with saline serving as the control. Ang II infusion lasted 4 weeks with the osmotic micropumps (Alzet, model 2004, Cupertino, CA), following previous description (Wang et al., 2020). Fourteen days after the subcutaneous implantation of micropump, blood pressure was measured to assess the efficacy of the model.

### 2.4 Grouping and treatment

All mice were randomly divided into six groups according to ear tagging using the random number table method. Saline control group (Con), HR model group (Ang II), HR model + low dose ZLHXTY (0.35 g/kg) group (ZL-LD), HR model + medium dose ZLHXTY (0.7 g/kg/day) group (ZL-MD), HR model + high dose ZLHXTY (1.4 g/kg/day) group (ZL-HD) (Mazhar et al., 2023), HR model + Enalapril Maleate Tablets (10 mg/kg/day) group (EMY) (Geng et al., 2023). ZLHXTY was administered by oral gavage beginning 1 day after HR induction, with daily dosing (200 μL) for 28 consecutive days at 08:00 a.m. Mice in Con and Ang II group received the same volume of saline. On 28th day, all mice were administered an overdose of pentobarbital by intraperitoneal injection for euthanization. The extracted retinal were preserved at −80°C for future analysis, while others were fixed with 10% paraformaldehyde for histological staining. Serum was collected and stored at −80°C.

### 2.5 Systolic blood pressure (SBP) measurement

SBP in varying groups were measured with the tail-cuff device (BP-2010A, Softron, Japan). Mice were placed in a metallic restrainer for 10 min before measurement. And SBP readings were taken once the waveform stabilized. A minimum of three measurements were recorded for each mouse. Baseline SBP was assessed 1 day before surgery, and subsequent measurements were taken on days 14, 21 and 28 following Ang II or saline infusion.

### 2.6 Electroretinography (ERG)

ERG tests (RetiMINER-P, IRC Medical Equipment, China) were performed on the mice at day 28th. After 24 h of dark acclimation, all mice were anesthetized appropriately and placed on an experimental table under weak red light. The two corneas were connected to different gold wire electrodes, together with the subcutaneous insertion of the common electrode into the tail, and the connection of reference electrode to the neck. We obtained the ERG recordings of the a-wave, b-wave, and Ops wave following flash stimuli of 0.01 and 3.0 cds/m^2^.

### 2.7 Optical coherence tomography angiography (OCTA)

Each animal was examined using ultra-widefield swept source OCTA (BM400K BMizar, TowardPi Medical Technology Ltd., China) after 28 days experiment. The mice were subjected to abdominal anesthesia, followed by pupil dilation using compound tropicamide eye drops. Next, the mice were gently positioned on the imaging platform. Automatic mode was selected to get a clear image, OCTA data was obtained. Furthermore, the vessel area density (VAD) and the vessel length density (VLD) were calculated automatically with the built-in software.

### 2.8 H&E and immunohistochemical (IHC) staining

After 72 h of immobilization in eye fixative, eyeball tissues underwent ethanol dehydration, xylene clearing, and paraffin-embedding. Subsequently, the 4 μm slices received H&E staining following standard protocols. For IHC staining, sections received the incubation of primary antibodies against Iba-1 (1:500) and Caspase 1 (1:100), and appropriate secondary antibodies. Images were captured with K-viewer software (version 1.5.3.1, KFBIO, Ningbo, China), at the optic nerve head 500 μm.

### 2.9 Enzyme-linked immunosorbent assay (ELISA)

ELISA kits was employed for measuring IL-6, IL-18, IL-1β serum levels as per the producer’s protocol.

### 2.10 Antioxidant activity assays

Homogenized retinal tissues were centrifuged to isolate the supernatants. The levels of MDA, GSH, GSH-px, SOD, and CAT in retinal tissues were quantified by virtue of commercially available kits, as per the producer’s protocol.

### 2.11 TUNEL staining

After dewaxing in xylene, paraffin sections underwent hydration through a graded alcohol series. The One Step TUNEL Apoptosis Assay Kit was adopted for retinal cell apoptosis as per the manufacturer’s protocol. Fluorescence was detected in 565 nm under fluorescence microscope (Leica DM4 B).

### 2.12 Immunofluorescence (IF) and DHE staining

Paraffin slices underwent antigen retrieval and 1 h of blockage in 5% BSA in succession. The slices received one night of incubation at 4°C using primary antibodies: NeuN Rabbit mAb (1:100), VEGFA (1:400), GFAP (1:100), TNF-α (1:200), NLRP3 (1:100), GSDMD (1:100), and another 2 h of incubation using secondary antibodies conjugated to Alexa594 (1:200) or Alexa488 (1:200) at room temperature. For DHE staining, the fresh frozen eyeball slices were with DHE for 30 min at RT. The last step was the 5 min of DAPI staining for nucleus. A fluorescence microscope (Leica DM4 B, Germany) was employed for capturing relevant images, and the Image J software (National Institutes of Health, United States) served for detecting the fluorescence intensity.

### 2.13 Western blot analysis

Retinal tissue samples underwent lysis treatment in RIPA buffer. BCA protein assay kit served for quantifying the protein concentration. After SDS-PAGE separation, proteins were moved to PVDF membranes. The blots received 1 h of blockage in 5% skimmed milk at RT and additionally one night of incubation using primary antibodies at 4°C: NF-κB p65 (1:1,000), TNF-α (1:1,000), IL-1β (1:1,000), and GAPDH (1:1,000). The following day, the blots underwent 1 h of incubation using horseradish peroxidase-labeled goat anti-rabbit IgG antibody (1:5,000) at room temperature. An enhanced chemiluminescence detection kit served for band visualization and the Tanon 5200 Automatic Chemiluminescence Imaging System (ChemiScope Capture, Shanghai, China) served for band analysis. Quantification was performed using ImageJ software.

### 2.14 Transmission electron microscopy (TEM)

After the execution of mice, the whole eyeballs underwent fixation treatment in 3% glutaraldehyde, and post-fixation in 1% osmium tetroxid. After dehydration via acetone solutions, the tissue underwent Epon 812 resin-embedding. A Leica UC7 ultramicrotome (Leica, Germany) was employed to obtain 60–90 nm ultrathin sections. Post-staining was performed for 10 min with 5% uranyl acetate, followed by 5 min of staining with Reynolds’ lead citrate. Examination of the prepared sections was conducted via a TEM (JEM-1400FLASH, Japan).

### 2.15 4D label-free quantitative proteomic analyses

Mouse retinal tissue received 4D-label-free quantitative proteomic analysis (Zhongke New Life Biotechnology, Shanghai, China). Briefly, SDT buffer (4% SDS, 100 mM Tris-HCl) was employed for protein extraction, and trypsin (MS Grade, Promega, United States) was employed for protein digestion as per the filter-aided sample preparation (FASP) protocol (Matthias Mann). After desalting treatment on C18 cartridges (Empore™ SPE, Sigma), the resulting peptides were reconstituted in 40 µL of 0.1% (v/v) formic acid (FA) after vacuum centrifugation. After being loaded onto an analytical C18-reversed phase column, peptides received linear gradient separation using buffer A (0.1% formic acid in water) and buffer B (84% acetonitrile, 0.1% FA) at 300 nL/min flow rate. Mass spectrometry adopted positive ion mode, with MS data obtained by virtue of a data-dependent top 20 method, engaging in the dynamic selection of the most plentiful precursor ions for HCD fragmentation (300–1,800 m/z). We set the automatic gain control (AGC) target to 1e6, with a max injection time of 50 ms and a dynamic exclusion duration of 30 s, followed by obtaining survey scans at 60,000 resolution at m/z 200, with HCD spectra acquired at 15,000 resolution at m/z 200 and an isolation width of 1.5 m/z. We set the normalized collision energy to 30 eV, and the underfill ratio as 0.1%. MaxQuant 1.6.14 software was adopted to process and search MA raw data to identify and quantify proteins.

### 2.16 Bioinformatic analyses

The NCBI BLAST + client software (ncbi-blast-2.2.28+-win32.exe) together with InterProScan were adopted for the local searching of the protein sequences pertaining to the identified DPEs, aiming at confirming homologous sequences. The procedure was followed by the assigning of GO terms, and the annotation of sequences by virtue of the Blast2GO software. Then, we queried the proteins from the KEGG database (http://geneontology.org/) for the acquisition of corresponding KEGG orthology identifiers, and mapped them to relevant biological pathways in KEGG.

### 2.17 Network pharmacology

Based on previous HPLCHR-MS results, 12 compounds in ZLHXTY target prediction were performed by HERB database (http://herb.ac.cn/) and Swiss target prediction database (http://www.swisstargetprediction.ch/). The Genecards database (https://www.genecards.org/) was utilized to screen for targets associated with disease, “hypertensive retinopathy” as the keyword. The intersecting targets between ZLHXTY and HR were acquired by drawing the Venn diagram (Venny2.1.0 software).

The composition of ZLHXTY and the intersecting targets were introduced into Cytoscape 3.10.1 to construct the “component-target-disease” interaction network, with different node colors representing components, target genes. The intersecting targets of ZLHXTY and HR were imported into STRING (https://string-db.org/) to construct a protein-protein interaction (PPI) network. With the confidence level set at ≥0.4, the results file in TSV format was downloaded and then imported into Cytoscape 3.10.2 software. The degree of each node was calculated using the “Tools-NetworkAnalyze” function. Core targets were selected based on three centrality metrics: betweenness centrality (BC), degree centrality (DC), and closeness centrality (CC). The intersecting targets of ZLHXTY and HR were imported into the DAVID database (http://david.nifcrf.gov/) for Gene Ontology (GO) functional enrichment analysis and Kyoto encyclopedia of genes and genomes (KEGG) pathway enrichment analysis.

### 2.18 Statistical analysis

Data presentation followed the means ± SEM format. Data analysis relied on GraphPad Prism 10.1.2 (GraphPad Software Inc., San Diego, United States). One-way ANOVA together with Tukey’s *post hoc* test assisted in assessing the group difference. P < 0.05 indicates statistical significance.

## 3 Results

### 3.1 Ang II infusion induces HR mice

**FIGURE 1:**
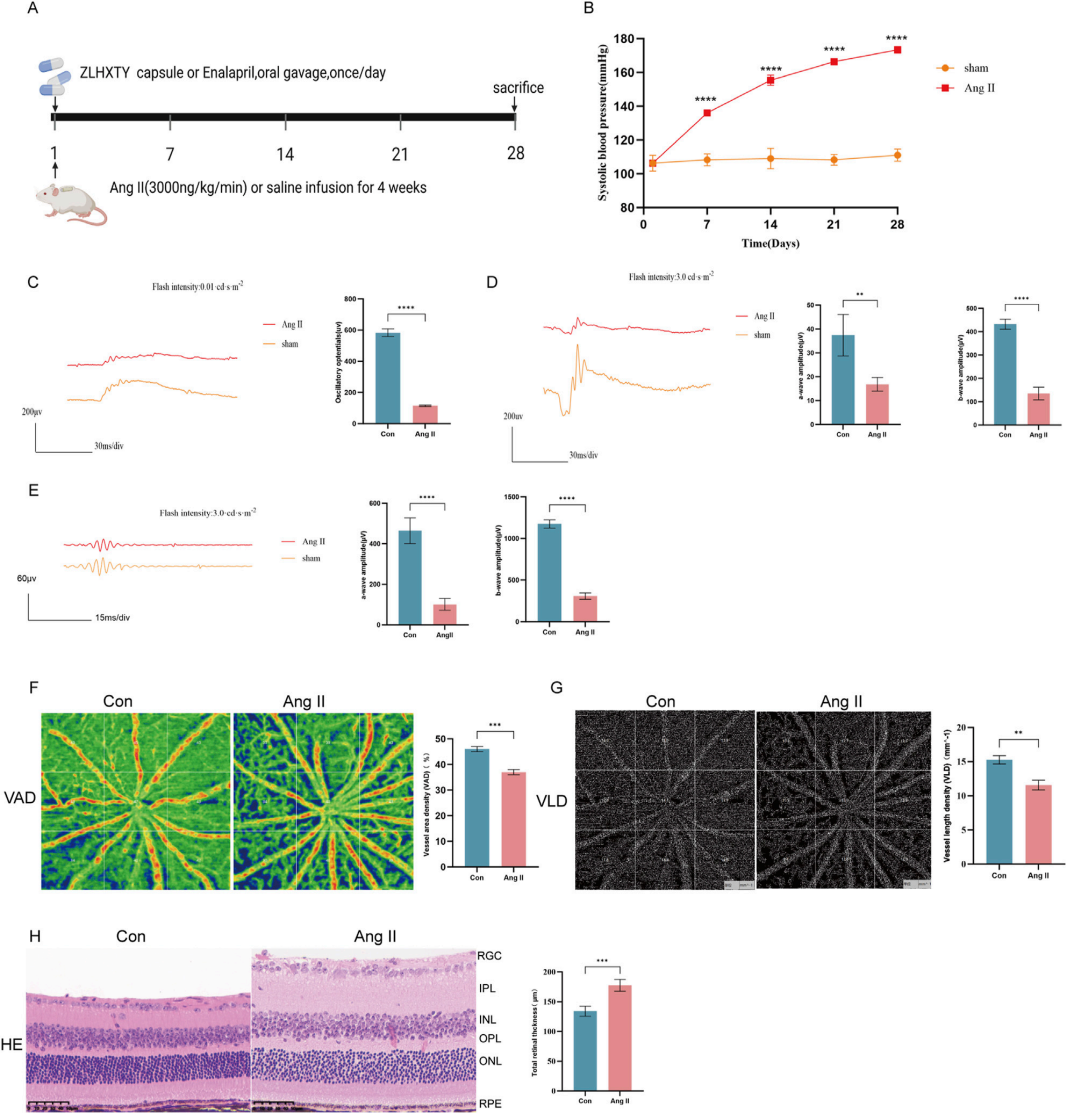
Subcutaneous implant micro-pump infusion of Ang II induces HR model in mice. **(A)** Schematic diagram of the experimental design. **(B)** The SBP levels of mice after infused with Ang II or saline for 14 21, and 28 days. **(C)** Wave forms of OPs (left) and the amplitude quantification. **(D)** Wave forms of a-wave and b-wave in flash stimulus of 0.01 cds/m^2^ and the amplitude quantification. **(E)** Wave forms of a-wave and b-wave in flash stimulus of 3.0 cds/m^2^ and the amplitude quantification. **(F)** Images of OCTA of the VAD (left) and the quantitative value. **(G)** Images of OCTA of the VLD (left) and the quantitative value. **(H)** Representative images of H&E staining of the retinal tissue (left) and quantitation of the retinal thickness (Scale bar: 50 μm). ***P < 0.001 and **P < 0.01 vs. the Con group.

We used a classic subcutaneous implant micro-pump infusion of Ang II to establish a mouse model of HR (Figure 1A). As expected, 2 weeks of Ang II infusion later, the SBP in mice reached 150 mmHg (Figure 1B), meeting the standard criteria for HR modeling. Retinal neurodegeneration is an early feature of HR, and oscillatory potentials (OPs) indicate the early-stage retinal circulation disorder. On day 28th, we performed ERG tests, that revealed significant reduction in a-wave, and b-wave amplitudes in different flash stimuli (Figures 1C,D), OPs (Figure 1E) in Ang II infused HR mice. Retinal vascular injury is another early feature of HR. To evaluate the vascular changes, we performed OCTA, that revealed significantly decreased VAD (Figure 1F), large area of blue region in Ang II group and VLD (Figure 1G) in Ang II infused mice. H&E staining revealed pathological changes in retina such as edema with disordered and sparse arrangement of the retinal cells after Ang II infusion in mice (Figure 1H). These findings collectively confirmed the successful establishment of the HR model.

### 3.2 ZLHXTY alleviates retinal dysfunction in HR mice

**FIGURE 2:**
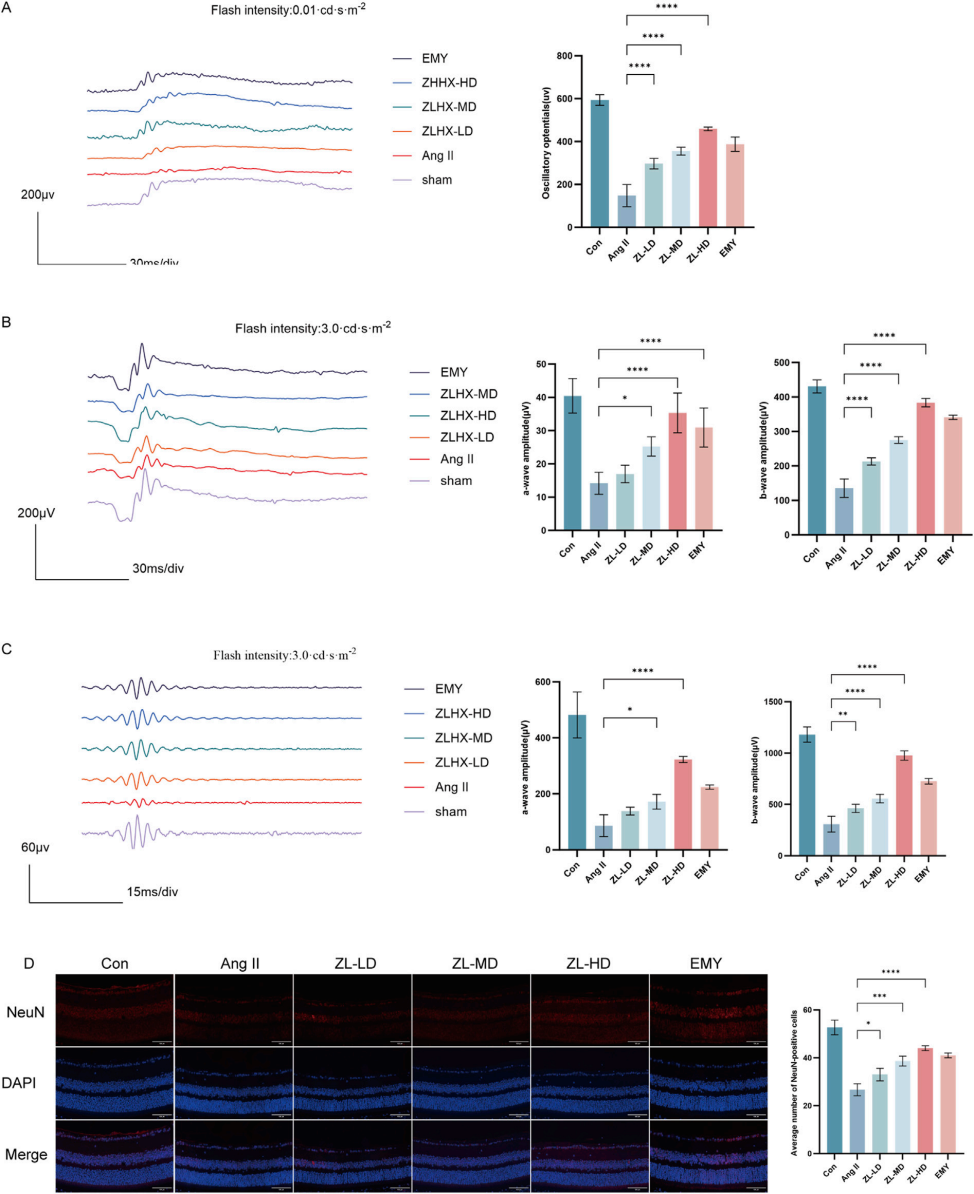
ZLHXTY alleviates retinal dysfunction in HR mice. **(A)** Wave forms of OPs (left) and the amplitude quantification. **(B)** Wave forms of a-wave and b-wave in flash stimulus of 0.01 cds/m^2^ (left) and the amplitude quantification. **(C)** Wave forms of a-wave and b-wave in flash stimulus of 3.0 cds/m^2^ (left) and the amplitude quantification. **(D)** Representative images of IF of NeuN (red) in retinal slices (left) and the quantification of IF intensity. ***P < 0.001 and **P < 0.01 *P < 0.05 vs. the Ang II group.

On day 28th, the effects of ZLHXTY on HR visual function were assessed by analysis of ERG changes. ERG tests clearly demonstrated that the decrease in both a-wave and b-wave amplitudes in different flash stimuli markedly rescued after treatment with ZLHXTY (Figures 2A,B). Consistent with this, the reduction in Ops amplitudes was also reversed after treatment with ZLHXTY (Figure 2C). Recent research indicates that retinal function is partially linked to retinal ganglion cells (RGCs) loss (Zeng et al., 2023). The Ang II Group exhibited obviously weaker cell density of NeuN + cells in RGCs versus the Con group and the ZLHXTY group (Figure 2D). Even the ZL-HD group had a better protection effect than EMY group. Overall, the above findings suggest that ZLHXTY alleviate retinal dysfunction by protecting the loss of RGCs in HR mice.

### 3.3 ZLHXTY can improve vascular injury in HR mice

**FIGURE 3:**
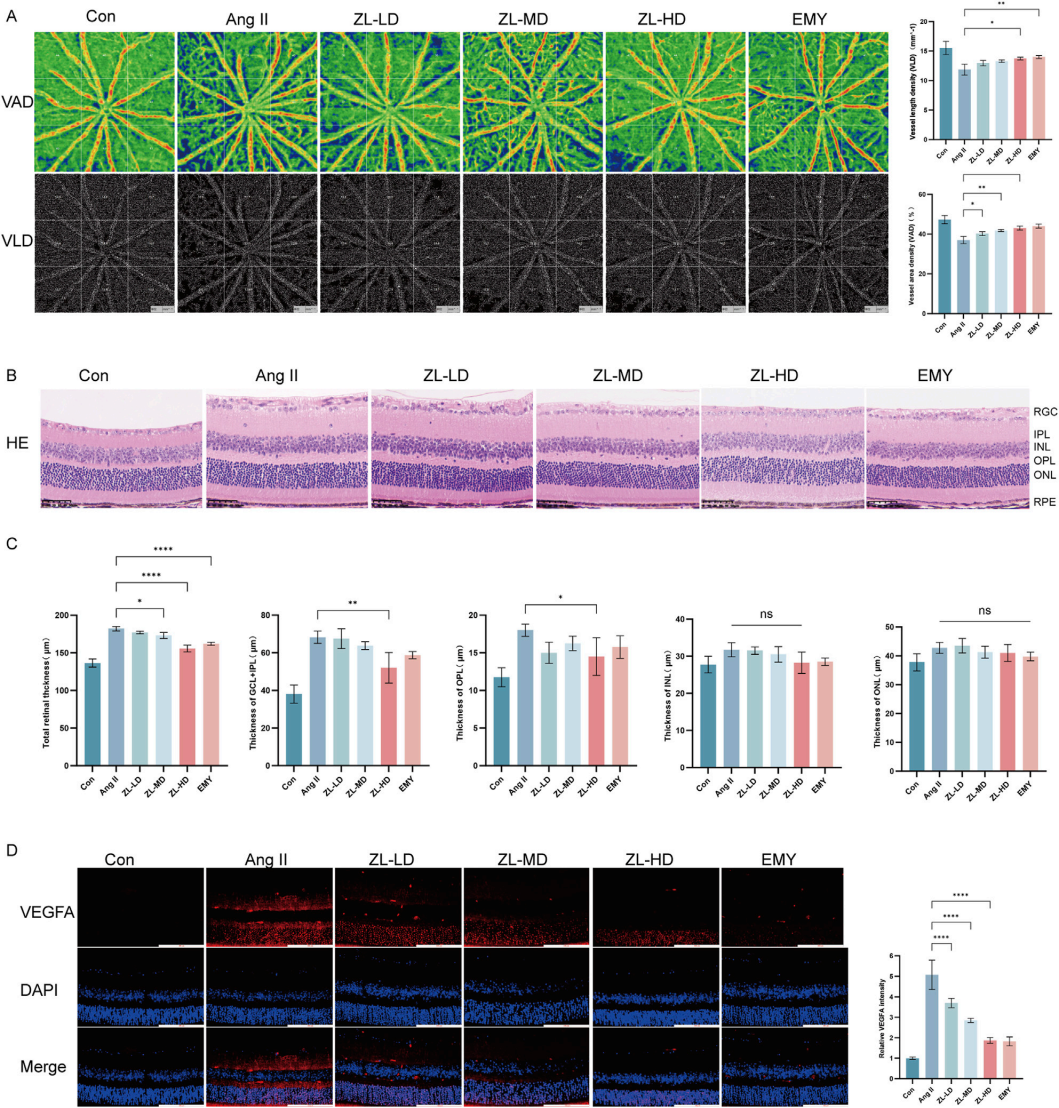
ZLHXTY can improve vascular injury in HR mice. **(A)** Images of OCTA of the VAD and VLD (left), and the quantitative value. **(B)** Representative images of HE staining of the retinal tissue (Scale bar: 50 μm). **(C)** Quantitation of the retinal thickness in different layers. **(D)** Representative images of IF of VEGFA (red) in retinal slices (left) and the quantification of the VEGFA fluorescence intensity (right, Scale bar: 100 μm). ***P < 0.001 and **P < 0.01, *P < 0.05 vs. the Ang II group.

According to OCTA, VAD and VLD in ZLHXTY group were significantly increased than in Ang II group (Figure 3A). In addition, HE staining also showed that ZLHXTY treatment significantly alleviated retinal structural damage, decreased central retinal thickness, notably the thickness of the ganglion cell layer (GCL) + inner plexiform layer (IPL) and outer plexiform layer (OPL), versus the Ang II group (Figures 3B,C). The increased vascular permeability critically marks the retinopathy, while VEGFA can essentially drive the vascular permeability (Uemura et al., 2021). The IF staining for VEGFA showed increased expression in retinal samples in Ang II group, but was reduced in the ZLHXTY treated mice (Figure 3D). Taken together, these results indicate that ZLHXTY can alleviate retinal vascular injury in HR mice by inhibiting VEGFA expression.

### 3.4 Network pharmacology analysis

**FIGURE 4:**
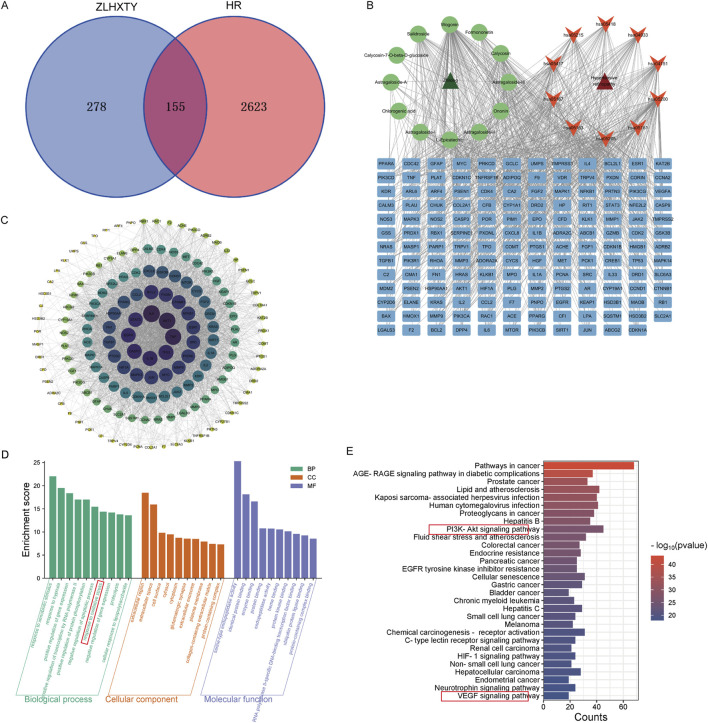
Network pharmacology of ZLHXTY alleviate HR. **(A)** Venn diagram of ZLHXTY composition targets and HR targets. **(B)** “compounds-targets-disease” network visualization. **(C)** PPI network of 155 integrating targets. **(D)** GO analysis of integrating targets. **(E)** KEGG analysis of integrating targets.

Based on the previous HPLCHR-MS results, a total of 433 targets were identified for 12 compounds of ZLHXTY. Meanwhile, 2,718 HR-related targets were obtained through the disease database. By integrating the HR-related targets and the ZLHXTY-related targets, 155 intersecting targets were successfully obtained (Figure 4A). To further explore the mechanism of ZLHXTY alleviate HR, we use Cytoscape 3.10.1 software to construct “component-target-disease” network, Degrees of freedom analysis revealed that Wogonin exhibited higher values than others among the active compounds (Figure 4B). In order to investigate the PPI among the 155 intersecting targets, a PPI analysis was carried out. Based on the betweenness BC, DC, and CC values, the top 10 core targets were identified, namely IL-6, AKT1, TNF, TP53, IL-1β, CASP3, EGFR, STAT3, BCL2, NF-κB1, as illustrated in Figure 4C. GO enrichment analysis revealed that intersecting targets were primarily involved in response to oxidative stress, response to hypoxia, extracellular region, serine-type endopeptidase activity (Figure 4D). Additionally, KEGG enrichment analysis emphasized the significant enrichment in pathways such as PI3K-Akt signaling pathyway, VEGF signaling pathway, HIF-1 signaling pathway (Figure 4E).

### 3.5 Mechanisms of ZLHXTY on HR in mice with proteomic analyses

**FIGURE 5:**
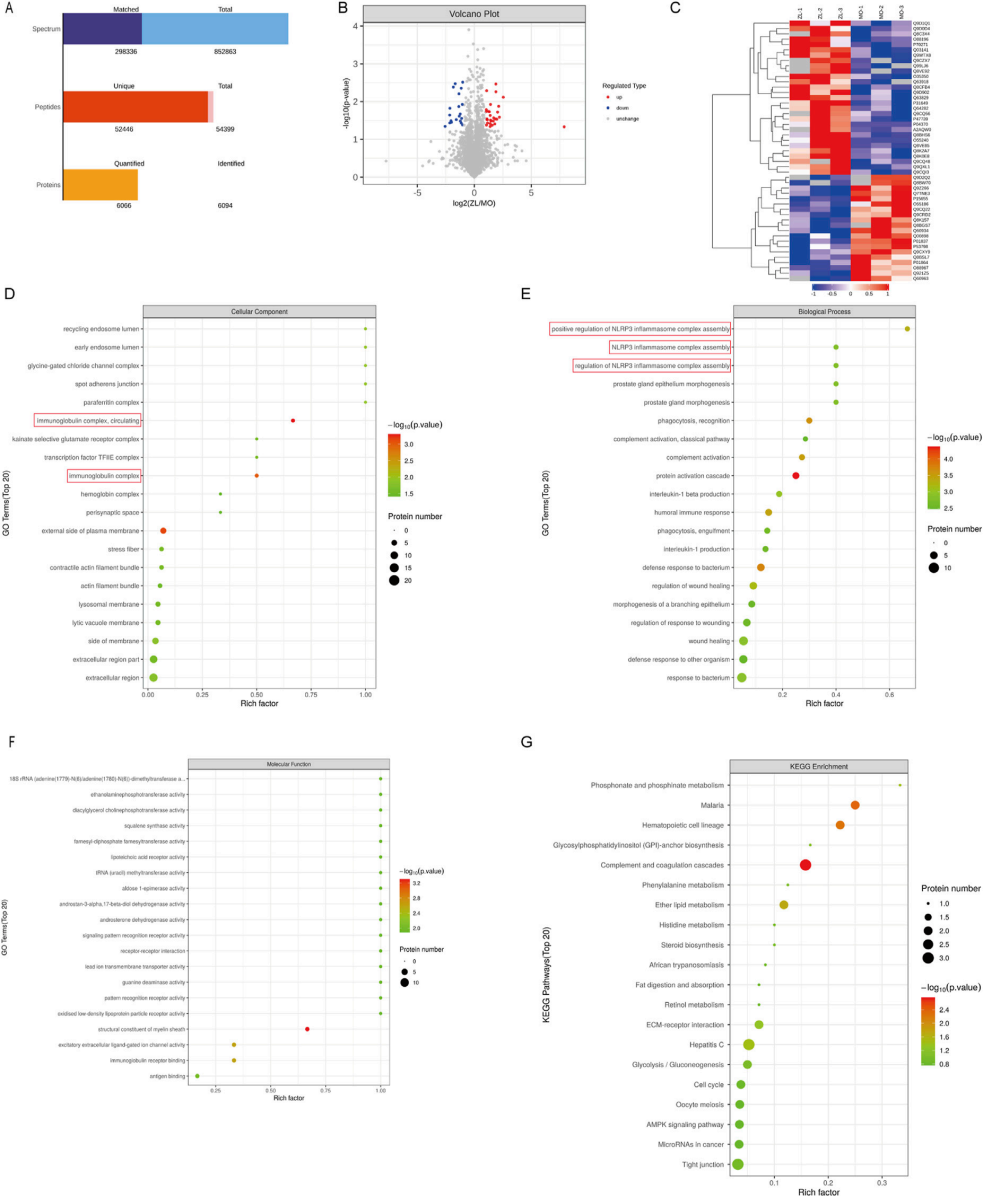
ZLHXTY on HR with proteomic analyses in mice. **(A)** The basic statistical results of MS. **(B)** Volcano plots of the DEPs. The red points represent proteins that have been significantly upregulated, the blue points reflect proteins that have been significantly downregulated, and the grey points show no protein quantitative information. **(C)** Hierarchical cluster analysis of DEPs. Annotation term levels of DEPs between Ang II group vs. ZLHXTY group are indicated as biological process **(D)**, cellular component **(E)**, molecular Function **(F)**, and KEGG pathway **(G)**.

To unravel the mechanism of ZLHXTY in HR, we adopted 4D label-free quantitative proteomic analysis to confirm differentially expressed proteins (DEPs) in the retina of Con, Ang II and ZL three groups. Considering the outcomes of animal experiments, the ZL-HD group underwent the proteomic studies. According to principal component analysis, these groups present remarkably different protein expressions. Based on these qualified data, total of 6,094 proteins were identified, 6,066 of them meeting the quantitative requirements (Figure 5A). Taking a fold change >2.0 times and p < 0.05 as thresholds, we identified 38 upregulated and 36 downregulated proteins in Ang II group versus Con group (Table 2). A volcano plot was drew to display these DEPs (Figure 5B). For achieving a better visualization of the protein abundance difference, DEPs were subjected to a hierarchical clustering analysis (Figure 5C). Specifically, different proteins in cluster 3 showed a clear upregulation in Ang II group, but an obvious downregulation in ZL group. GO and KEGG pathway analyses reveal the biological and function pathways pertaining to DEPs. Three sets of ontologies, i.e., biological process (BP), cellular component (CC), and molecular function (MF) were adopted for the individual analysis of the DEPs. In Figures 5D–F, the DEGs in MF were in “immunoglobulin receptor binding”, those in CC were in “immunoglobulin complex, circulating”, “immunoglobulin complex”, and those in BP were in “protein activation cascade”, “phagocytosis, recognition”, “humoral immune response”, “positive regulation of NLRP3 inflammasome complex assembly”, “interleukin-1 beta production”, “regulation of NLRP3 inflammasome complex assembly”, “NLRP3 inflammasome complex assembly”, “phagocytosis, engulfment”, “interleukin-1 production”. Moreover, the KEGG pathway analysis demonstrates the principal involvement of the DEPs in complement and coagulation cascades, and AMPK signaling pathway (Figure 5G). Thus, in summary, the mechanism of ZLHXTY treatment of HR may be related to immune-inflammatory regulation and oxidative stress, and activation of NLRP3 inflammasome may be the main mechanism.

**TABLE 2:** Identification differentially expressed proteins of Ang II group and ZL group.

Gene Name	Protein ID	Protein Name
up-regulated proteins		
Mbp	P04370	Myelin basic protein
Rbm4b	Q8VE92	RNA-binding protein 4B
Kif21b	Q9QXL1	Kinesin-like protein KIF21B
Slc6a13	P31649	Sodium- and chloride-dependent GABA transporter 2
Gpx7	Q99LJ6	Glutathione peroxidase 7
Gbp5	Q8CFB4	Guanylate-binding protein 5
Pip4p2	Q9CZX7	Type 2 phosphatidylinositol 4,5-bisphosphate 4-phosphatase
Mark3	Q03141	MAP/microtubule affinity-regulating kinase 3
Guf1	Q8C3X4	Translation factor Guf1, mitochondrial
Nudcd2	Q9CQ48	NudC domain-containing protein 2
Cavin2	Q63918	Caveolae-associated protein 2
Use1	Q9CQ56	Vesicle transport protein USE1
Armcx3	Q8BHS6	Armadillo repeat-containing X-linked protein 3
Ttc3	O88196	E3 ubiquitin-protein ligase TTC3
Aldh3a1	P47739	Aldehyde dehydrogenase, dimeric NADP-preferring
Capn1	O35350	Calpain-1 catalytic subunit
Commd3	Q63829	COMM domain-containing protein 3
Prelid3a	Q8VE85	PRELI domain containing protein 3A
Mphosph6	Q9D1Q1	M-phase phosphoprotein 6
Map3k15	A2AQW0	Mitogen-activated protein kinase kinase kinase 15
Gtf2e2	Q9D902	General transcription factor IIE subunit 2
Ints10	Q8K2A7	Integrator complex subunit 10
Dimt1	Q9D0D4	Probable dimethyladenosine transferase
Pdlim4	P70271	PDZ and LIM domain protein 4
Ifit1	Q64282	Interferon-induced protein with tetratricopeptide repeats 1
Mad1l1	Q9WTX8	Mitotic spindle assembly checkpoint protein MAD1
Rdh5	O55240	Retinol dehydrogenase 5
Gmfb	Q9CQI3	Glia maturation factor beta
Fgb	Q8K0E8	Fibrinogen beta chain
down-regulated proteins		
Usp38	Q8BW70	Ubiquitin carboxyl-terminal hydrolase 38
Fdft1	P53798	Squalene synthase
Snapin	Q9Z266	SNARE-associated protein Snapin
Lamtor1	Q9CQ22	Ragulator complex protein LAMTOR1
Spag7	Q7TNE3	Sperm-associated antigen 7
Arf2	Q8BSL7	ADP-ribosylation factor 2
Cd59a	O55186	CD59A glycoprotein
Serpina1e	Q00898	Alpha-1-antitrypsin 1-5
Pigk	Q9CXY9	GPI-anchor transamidase
Grik1	Q60934	Glutamate receptor ionotropic, kainate 1
Trmt44	Q9D2Q2	Probable tRNA (uracil-O(2)-)-methyltransferase
Pla2g7	Q60963	Platelet-activating factor acetylhydrolase
Igkc	P01837	Immunoglobulin kappa constant
Fgf2	P15655	Fibroblast growth factor 2
Yme1l1	O88967	ATP-dependent zinc metalloprotease YME1L1
Emc2	Q9CRD2	ER membrane protein complex subunit 2
Cept1	Q8BGS7	Choline/ethanolaminephosphotransferase 1
	P01864	Ig gamma-2A chain C region secreted form
Galm	Q8K157	Galactose mutarotase
Tnfaip8	Q921Z5	Tumor necrosis factor alpha-induced protein 8

### 3.6 ZLHXTY resist oxidative stress in HR mice

**FIGURE 6:**
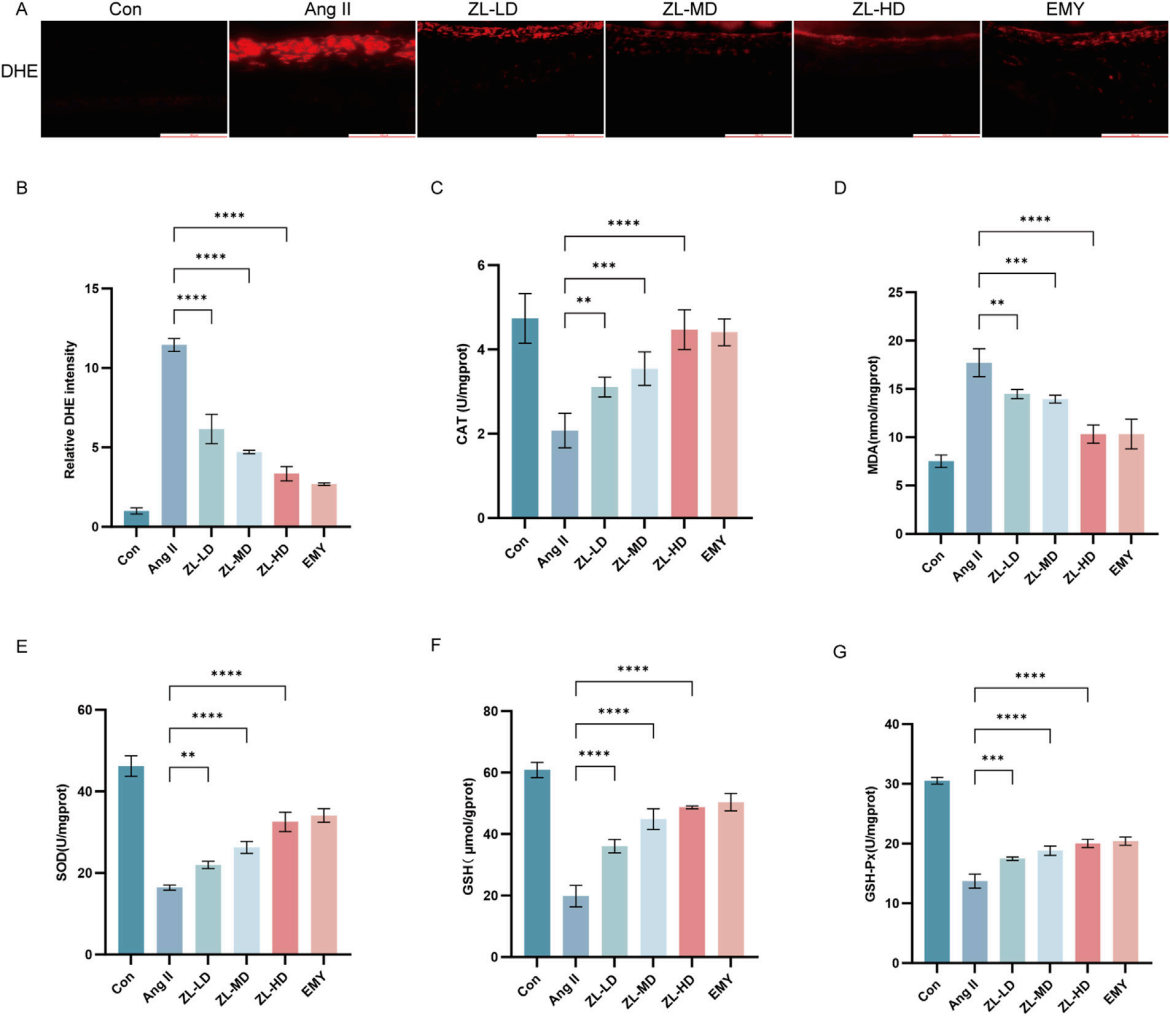
ZLHXTY resist the oxidative stress in HR mice. **(A)** Representative images of IF of DHE (red) in retina slices. **(B)** The red fluorescence intensity of DHE was quantified. **(C)** The levels of MDA in retina tissue. **(D)** The levels of SOD in retina tissue. **(E)** The levels of CAT in retina tissue. **(F)** The levels of GSH in retina tissue. **(G)** The levels of GSH-px in retina tissue. ***P < 0.001 and **P < 0.01 vs. the Ang II group.

Given the crucial role of oxidative stress in HR (Wan et al., 2023), and the previous network pharmacological results also suggest that oxidative stress is involved in the mechanism of ZLHXTY therapy for HR. We adopted DHE staining for the assessment of reactive oxygen species (ROS) levels in retina. The Ang II group exhibited higher ROS level versus the Con group (p < 0.001), whereas, the ZLHXTY significantly reduced ROS levels (p < 0.001) (Figure 6A,B). Furthermore, the Ang II group exhibited considerably higher serum levels of MDA versus Con group (Figure 6C). Conversely, SOD, CAT, GSH-Px and GSH levels were significantly decreased (Figures 6D–G). ZLHXTY treatment effectively reversed these effects in all five indicators. These findings suggest that ZLHXTY alleviates oxidative stress in the HR by enhancing antioxidant enzyme activity and reducing peroxide accumulation.

### 3.7 ZLHXTY inhibit inflammation in HR mice

**FIGURE 7:**
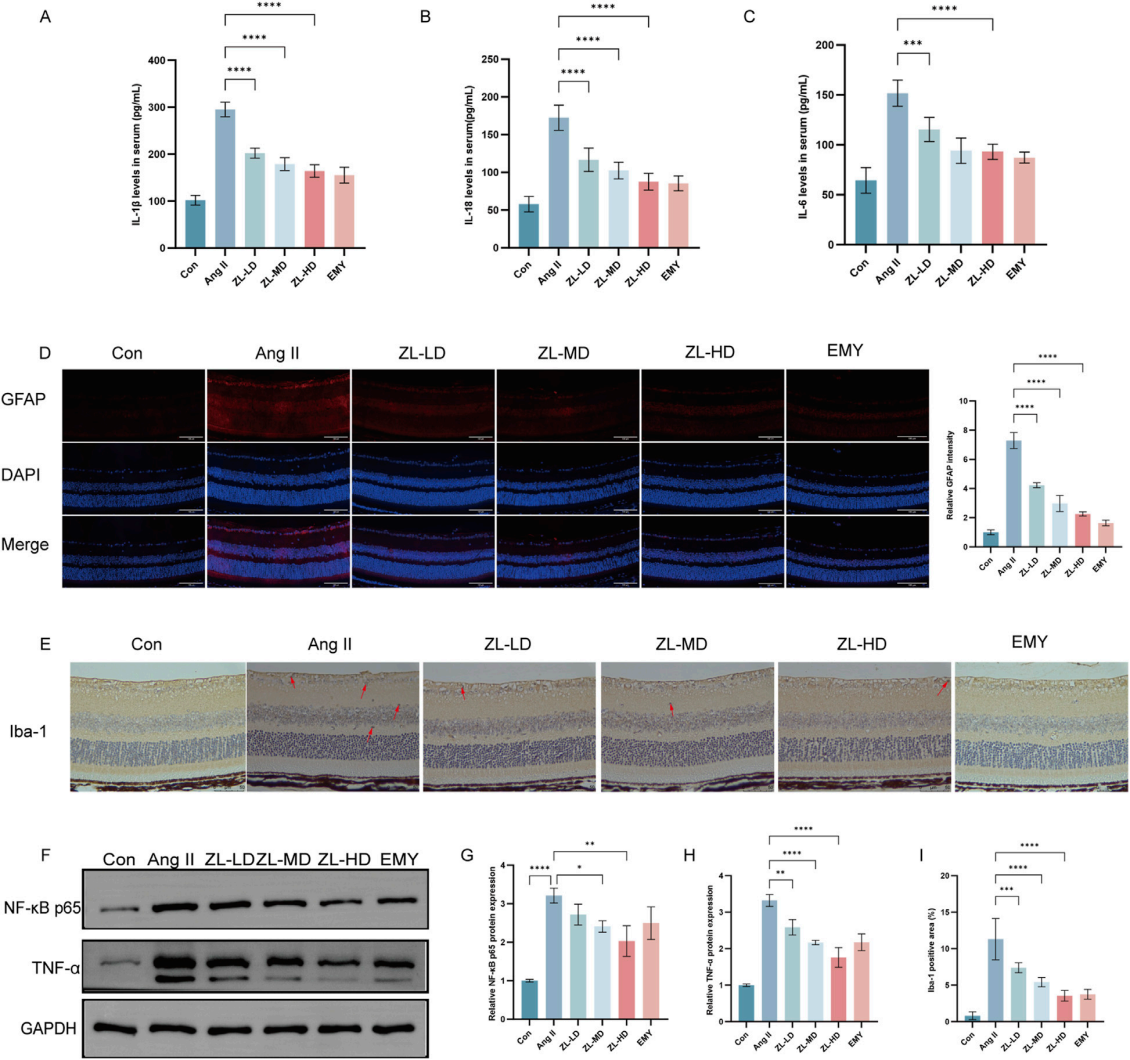
ZLHXTY inhibit inflammation in HR mice. **(A)** The levels of IL-1β in serum. **(B)** The levels of IL-18 in serum. **(C)** The levels of IL-6 in serum. **(D)** Representative images of IF of GFAP (red) in retinal slices (left) and the quantification of the red fluorescence intensity in each group (right, scale bar: 100 μm). **(E)** Representative images of Iba1 IHC staining, red arrows indicate the Iba1-positivemicroglia (scale bar: 50 μm, left). **(F)** The expression of NF-κB P65, TNF-α protein was analysed by Western blot in each group. **(G)** The NF-κB P65 protein levels was quantified. GAPDH was used as the internal control. **(H)** The TNF-α protein levels were quantified. GAPDH was used as the internal control. **(I)** Quantification of Iba1 positive cells in each group. ***P < 0.001 and **P < 0.01,*P < 0.05 vs. the Ang II group.

Inflammation also plays a vital role in HR (Yue and Zhao, 2020). With the aim of confirming the anti-inflammatory effect of ZLHXTY, we adopted ELISA kits for examining the serum levels of IL-6, IL-18, and IL-1β. According to Figures 7A–C, Ang II group exhibited remarkably elevated IL-1β, IL-18 and IL-6 levels versus Con group (p < 0.0001). Nevertheless, after ZLHXTY treatment, their levels were reduced (p < 0.0001). Müller cells and microglia, as the retina’s resident immune cells, crucially coordinate the inflammatory response (Wang et al., 2022). To further elucidate the ZLHXTY intervention mechanism in HR, we performed IF. The results presented that GFAP express primarily localized to the inner limiting membrane and retinal nerve fiber layer in Con group. However, in Ang II group, GFAP expression was significantly increased and distributed throughout the retinas. In contrast, GFAP expression was reduced in the ZL-treated group (Figure 7D). According to Iba-1 IHC results, macrophages were activated in Ang II group versus Con group, while there were less macrophages in ZL-treated mice (Figure 7E,I). Network pharmacological results suggest that NF-κB, TNF-α are core target protein of ZLHXTY for HR treatment. The results suggested that ZLHXTY suppressed TNF-α, and NF-κB p65 expression (Figure 7F–H). These results conclude that ZLHXTY could suppresses glial cell activation and the level of inflammation in HR mice.

### 3.8 ZLHXTY alleviates pyroptosis through NLRP3 inflammasome activation inhibition in HR mice

**FIGURE 8:**
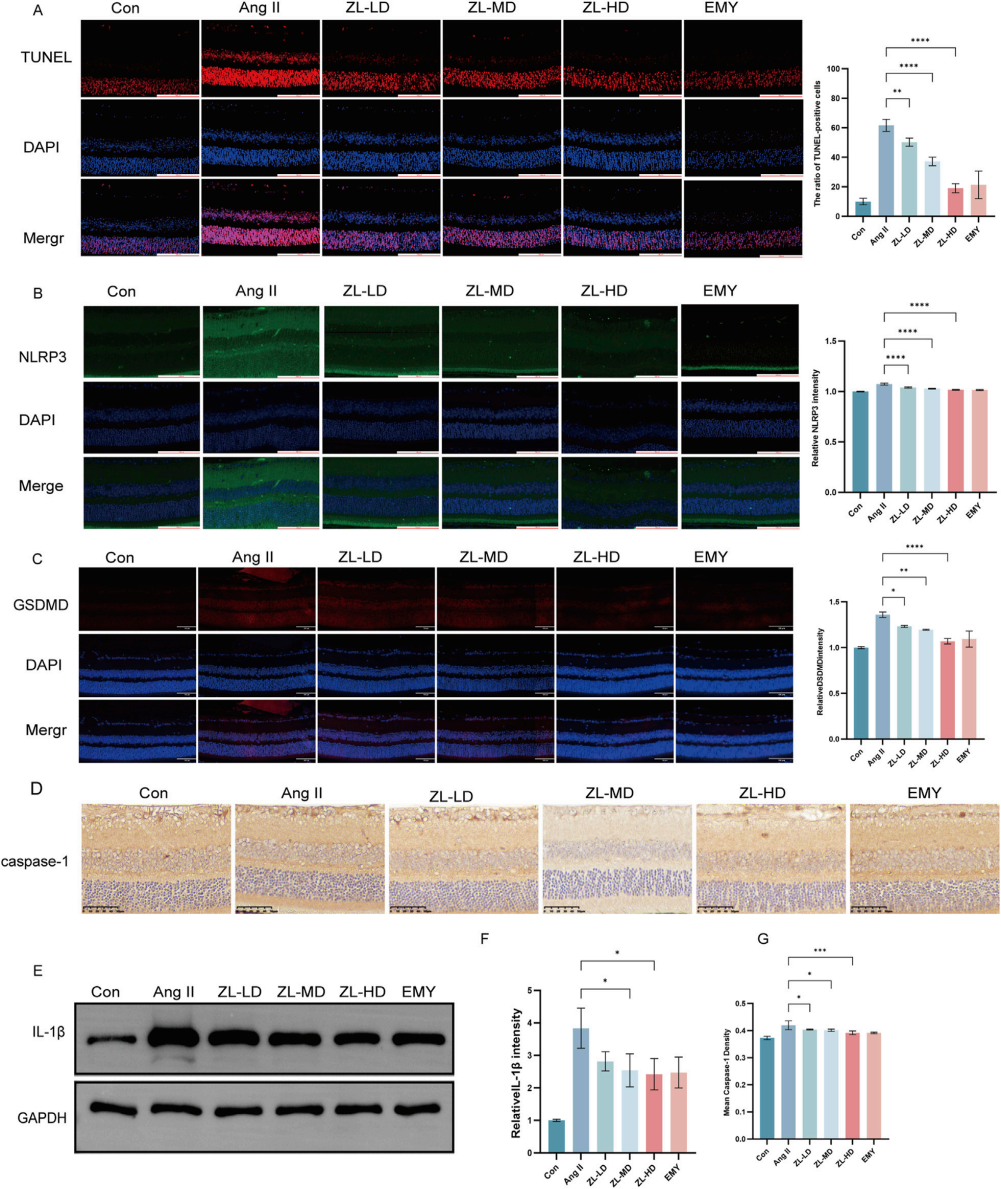
ZLHXTY alleviate apoptosis by inhabit NLRP3 inflammasome activation in HR mice. **(A)** Representative images of TUNEL (red) in retinal slices (left), and the ration of TUNEL positive cells in each group. **(B)** Representative images of IF of NLRP3 (green) in slices (left), and the quantification of the NLRP3 fluorescence intensity. **(C)** Representative images of IF of GSDMD (red) in retinal slices (left), and the quantification of the GSDMD fluorescence intensity in each group. **(D)** Representative images of IHC of caspase-1. **(E)** The expression of IL-1β protein was analysed by Western blot in each group. **(F)** The protein levels was quantified β-Action was used as the internal contro. **(G)** The quantification of mean of cleaved caspase-1 density. Scale bar = 100 μm.***P < 0.0001, and **P < 0.01,*P < 0.05 vs. the Ang II group.

**FIGURE 9:**
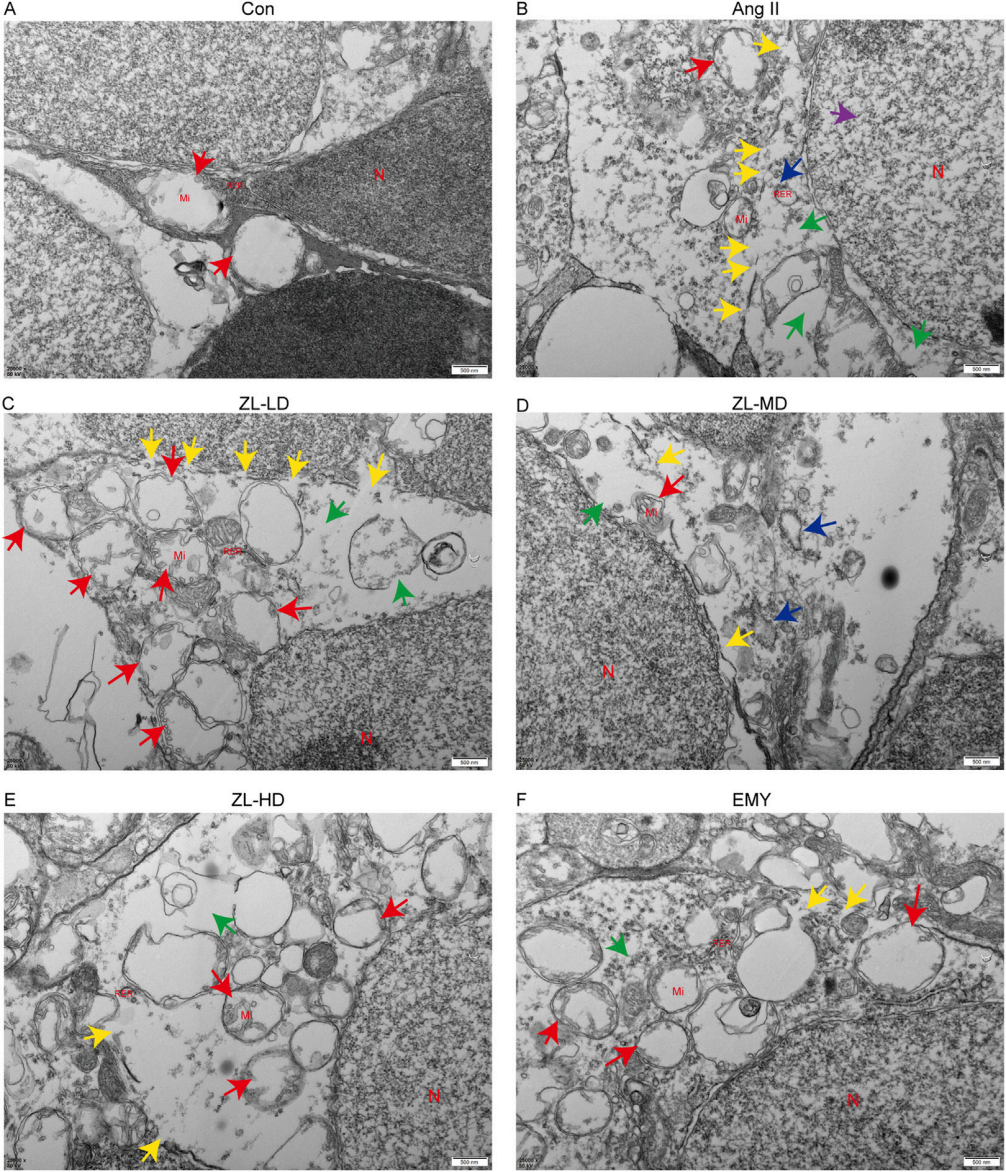
ZLHXTY alleviate apoptosis by inhabit NLRP3 inflammasome activation in HR mice. **(A–F)** Representative images of TEM in each group. N indicate cell nucleus, Mi indicate mitochondria, RER indicate rough endoplasmic reticulum, green arrows indicate ribosome loss, red arrows indicate mitochondrial swelling, purple arrows indicate the perinuclear space widened, blue arrows indicate trachyplasmic reticulum dilatation, yellow arrows indicate cell membrane discontinuity.

According to previous studies reported NLRP3 inflammasome involvement in pyroptosis. To investigate this, we employed TUNEL staining, ascertaining more positive cells in Ang II group versus Con group (p < 0.0001), while ZLHXTY treatment reduced the number of positive cells (Figure 8A). Through Western blot and IF analysis (Figures 8B–E), NLRP3, caspase-1, IL-1β, and pyroptosis executor GSDMD presented strikingly increased protein levels versus the Con groups, whereas ZLHXTY markedly repressed the aforementioned alterations. Finally, TEM revealed that in Ang II group, many neurons showed signs of pyroptosis, with slight widening of the perinuclear space, severe loss of ribosomes in the cytoplasm, swelling and vacuolization of mitochondria, and partial expansion of the rough endoplasmic reticulum. Compared with Ang II group, there was minimal ribosomal loss in the cytoplasm, mild mitochondrial swelling, and only a few areas of discontinuity in the cell membrane in ZLHXTY group (Figures 9A–F). These findings illustrate that ZLHXTY can alleviate pyroptosis by inhibiting NLRP3 inflammasome activation.

## 4 Discussion

Since vascular remodeling is one of the most common signs of hypertensive organ damage, thus typical vascular injury is also among the first manifestations of HR (Wang et al., 2022). The blood-retinal barrier plays a crucial role in maintain retinal integrity and can be affected by various factors (Antonetti et al., 2021). VEGFA is a key factor contributing to blood - retinal barrier dysfunction (Dragoni and Turowski, 2018). Our study confirmed that ZLHXTY can increase retinal blood flow in HR mice. We also found that ZLHXTY can reduce the expression of VEGFA, thereby reducing vessel leakage. With the development of neurophysiological examination techniques, an increasing evidence demonstrates that retinal neurodegeneration occurs earlier than microvascular damage (Oshitari, 2021). Extensive studies have shown that in a variety of retinal diseases, the increased loss of RGCs occurs prior to microvascular injury. Moreover, the loss of RGCs is a crucial event in the process of retinal neurodegeneration (Wu and Mo, 2023). The hypertension milieu can cause the loss of RGCs and visual information cannot be transmitted (Farouk et al., 2021). Our results showed that ZLHXTY could alleviate the retinal neurodegeneration in HR mice by inhibiting the loss of RGCs.

Existing researches has confirmed that inflammation is a factor driving the development and progression of HR. During the pathological process of HR, tdue to blood exudation, lipid changes, and other factors, the retina generates a large number of inflammatory and chemotactic factors. These factors promote the migration of immune cells and trigger their excessive activation (Yue and Zhao, 2020). Activated immune cells further release inflammatory mediators and pro-inflammatory factors, thereby exacerbating cellular damage (Zhao B. et al., 2024). Müller cell is the only cell type that spans the entire retina. Their activation is manifested by upregulated GFAP expression (Li et al., 2025). Previous studies have shown that hypertension can induce the activation of Müller cell. In the physiological state, microglial cell is in a resting state, while in the early stage of HR, it will rapidly activate and migrate to the outer nuclear layer, release inflammatory factors and triggering immune inflammatory response (Padovani-Claudio et al., 2023). In our study, Ang II infusion stimulates Müller cells and microglia to be activated, and promotes inflammatory mediators (IL-18, IL-6, IL-1β, NF-κB, and TNF-α) to be more secreted. Treatment with ZLHXTY reversed these changes.

Oxidative stress occurs when there is an imbalance between the formation and removal of free radicals. The retina, with its high metabolic activity, is vulnerable to oxidative stress (Yang et al., 2024). According to previous studies, ROS levels increase significantly in the retina, resulting in severe oxidative stress damage (Wang et al., 2020). In our study, Ang II infusion induced ROS accumulation and made antioxidant enzymes (CAT, SOD, and GSH-Px) less activated, while increasing levels of MDA peroxidation. However, treatment with ZLHXTY prevented these alterations. Moreover, both in network pharmacology and proteomics results suggest that the mechanism of ZLHXTY alleviate HR is closely related to inflammation and oxidative stress.

NLRP3 inflammasome plays a crucial role in triggering the inflammatory response in retinal disease. Its activation consists of two main steps: priming and (Sun et al., 2024). The priming step takes place, when Toll-like receptors detect exogenous pathogens or endogenous danger signals. This stimulus causes NF-κB to enter the nucleus and transcription occurs, which then upregulates the expression of NLRP3 and IL-1β (He et al., 2016). Although existing researches have not fully elucidated the exact mechanism of NLRP3 inflammasome activation, extensive research have shown tha it is closely associated with ROS (Kuo et al., 2022). The abnormal accumulation of ROS promotes thioredoxin-interacting protein (TXNIP) to be dissociated from thioredoxin, enhancing the interaction between TXNIP and NLRP3, thus promoting NLRP3 inflammasome assembly (Fu and Wu, 2023). In recent studies, it has been found that NLRP3 expression is upregulated in HR mice, and the inhibition of NLRP3 can reduce the secretion of pro-inflammatory cytokines (Li J. et al., 2024). In our study, the expression of NLRP3 showed a marked increase after the infusion of Ang II. Conversely, upon treatment with ZLHXTY, there was a discernible decrease in NLRP3 expression. Moreover, the findings from our proteomics analysis strongly indicated that ZLHXTY predominantly exerts its regulatory effects on the NLRP3 inflammasome. Given these observations, we hypothesize that ZLHXTY mitigates the manifestations of HR by modulating the activity and function of the NLRP3 inflammasome. Recent accumulating evidence suggests that NLRP3/Caspase-1/GSDMD axis is the classic pyroptosis pathway (Gan et al., 2020). Another study has also revealed that retinal disease progresses with pyroptosis as an incipient symptom, and regulating pyroptosis can mitigate early retinal disease (Zhao et al., 2021). Our subsequent experimental results indicated that Ang II infusion effectively activates and highly upregulates the key components of the NLRP3 inflammasome complex, namely NLRP3, caspase-1, and GSDMD. However, after treatment with ZLHXTY, the expression levels of these components decreased. Finally, TEM results also showed that ZLHXTY treatment significantly alleviated pyroptosis in HR mice. Thus, the NLRP3/Caspase-1/GSDMD axis may be the molecular mechanism underlying the therapeutic effects of ZLHXTY in HR.

In conclusion, although ZLHXTY has been used in clinical treatment of hypertension-related complications for many years, existing researches have not clearly revealed the mechanism of HR. In this study, we are the first to identify ZLHXTY alleviates retinal neurodegeneration and vascular injury by inhibited pyroptosis via mediating NLRP3 inflammasome activation in HR mice. Our findings assist in understanding its potential as a target drug for HR prevention and treatment from new perspectives. However, the study has many limitations. First, we focused primarily on the effects of ZLHXTY on glial cells, neglecting other cell types. Therefore, further studies shall directly illustrate the therapeutic efficacy of ZLHXTY on other cells. Second, it has been demonstrated that ZLHXTY could blocked pyroptosis in HR mice, but the specific mechanism for such anti-apoptotic effects was not elucidated.

## 5 Conclusion

**FIGURE 10:**
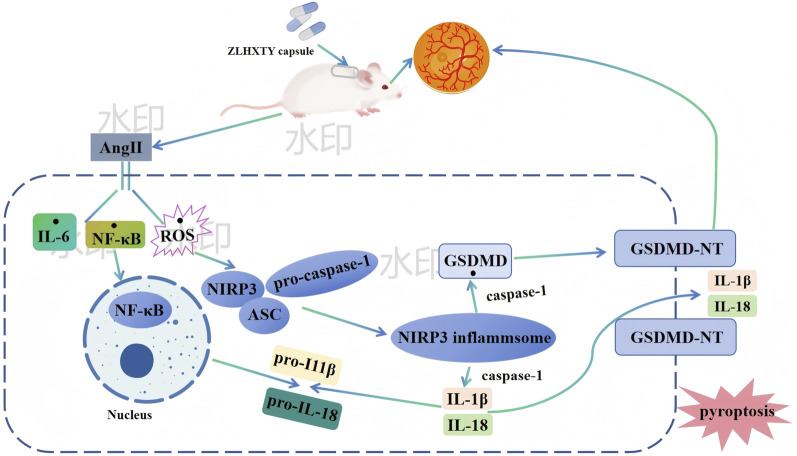
Possible mechanism underlying ZLHXTY alleviate HR.

In conclusion, based on the findings of our study, treatment with ZLHXTY effectively alleviated retinal dysfunction and vascular injury in HR mice by inhibiting oxidative stress and inflammation in the retinal tissues. The mechanism is likely associated with the regulation of the NLRP3/Caspase-1/GSDMD axis. Specifically, ZLHXTY alleviates pyrodeath by inhibiting the activation of the NLRP3 inflammasome Figure 10. These results suggest that ZLHXTY represents a promising therapeutic approach for the management of HR.

## Data Availability

The data presented in the study are deposited in the ProteomeXchange Consortium (https://proteomecentral.proteomexchange.org), accession number PXD059024.

## Abbreviations

HR, Hypertensive retinopathy; NLRP3, NOD-like receptor pyrin domain containing 3; ZLHXTY, Zhilong Huoxue Tongyu Capsule; Ang II, Angiotensin II; SBP, Systolic blood pressure; ERG, Electroretinography; OCTA, Optical coherence tomography angiography; VAD, vessel area density; VLD, vessel length density; TUNEL, Terminal deoxynucleotidyl transferase mediated nick end labeling; TEM, Transmission electron microscopy; OPs, oscillatory potentials; RGCs, loss of retinal ganglion cells; GCL, ganglion cell layer; IPL, inner plexiform layer; OPL, outer plexiform layer; ROS, reactive oxygen species; DEPs, differentially expressed proteins; BP, biological process; CC, cellular component; MF, molecular function.
